# Rapid Identification of Emerging Pathogens: Coronavirus

**DOI:** 10.3201/eid1103.040629

**Published:** 2005-03

**Authors:** Rangarajan Sampath, Steven A. Hofstadler, Lawrence B. Blyn, Mark W. Eshoo, Thomas A. Hall, Christian Massire, Harold M. Levene, James C. Hannis, Patina M. Harrell, Benjamin Neuman, Michael J. Buchmeier, Yun Jiang, Raymond Ranken, Jared J. Drader, Vivek Samant, Richard H. Griffey, John A. McNeil, Stanley T. Crooke, David J. Ecker

**Affiliations:** * Ibis Therapeutics, Carlsbad, California, USA; † The Scripps Research Institute, La Jolla, California, USA

**Keywords:** PCR, molecular epidemiology, emerging pathogens, SARS virus, infectious disease surveillance, ESI mass spectrometry, research

## Abstract

We describe a new approach for infectious disease surveillance that facilitates rapid identification of known and emerging pathogens. The process uses broad-range polymerase chain reaction (PCR) to amplify nucleic acid targets from large groupings of organisms, electrospray ionization mass spectrometry for accurate mass measurements of PCR products, and base composition signature analysis to identify organisms in a sample. We demonstrate this principle by using 14 isolates of 9 diverse *Coronavirus* spp., including the severe acute respiratory syndrome–associated coronavirus (SARS-CoV). We show that this method could identify and distinguish between SARS and other known CoV, including the human CoV 229E and OC43, individually and in a mixture of all 3 human viruses. The sensitivity of detection, measured by using titered SARS-CoV spiked into human serum, was ≈1 PFU/mL. This approach, applicable to the surveillance of bacterial, viral, fungal, or protozoal pathogens, is capable of automated analysis of >900 PCR reactions per day.

## Abstract 2

New surveillance approach can analyze >900 polymerase chain reactions per day.

Nucleic acid tests for infectious diseases are primarily based on amplification methods that use primers and probes designed to detect specific organisms. Because prior knowledge of nucleic acid sequence information is required to develop these tests, they are not able to identify unanticipated, newly emergent, or previously unknown infectious organisms. Thus, the discovery of new infectious organisms still relies largely on culture methods and microscopy, which were as important in the recent identification of the severe acute respiratory syndrome–associated coronavirus (SARS-CoV) as they were in the discovery of HIV 2 decades ago (*1*–*4*).

Broad-range polymerase chain reaction (PCR) methods provide an alternative to single-agent tests. By amplifying gene targets conserved across groups of organisms, broad-range PCR has the potential to generate amplification products across entire genera, families, or as with bacteria, an entire domain of life. This strategy has been successfully used with consensus 16S ribosomal RNA primers for determining bacterial diversity, both in environmental samples (*5*) and in natural human flora (*6*). Broad-range priming has also been described for detection of several viral families, including CoV (*7*), enteroviruses (*8*), retroid viruses (*9*), and adenoviruses (*10*). The drawback of this approach for epidemiologic applications is that the analysis of PCR products for mixed amplified samples requires sequencing hundreds of colonies per reaction, which is impractical to perform rapidly or on large numbers of samples. New approaches to the parallel detection of multiple infectious agents include multiplexed PCR methods (*11*,*12*) and microarray strategies (*13*–*15*). Microarray strategies are promising because undiscovered organisms might be detected by hybridization to probes designed to conserved regions of known families of bacteria and viruses.

We present an alternative approach for rapid, sensitive, and high-throughput detection of infectious organisms. We use broad-range PCR to generate amplification products from the broadest possible grouping of organisms, followed by electrospray ionization mass spectrometry and base composition analysis of the products (*16*,*17*). The base compositions of strategically selected regions of the genome are used to identify and distinguish organisms in the sample. Enhanced breadth of priming is achieved through the use of primers and probes containing 5-propynyl deoxycytidine and deoxyuridine nucleotides that offer increased affinity and base pairing selectivity (*18*,*19*). Positioning the 5-propynyl primidine-modified nucleotides at highly conserved positions enables priming at short consensus regions and significantly increases the extent to which broad groups of organisms can be amplified.

## Materials and Methods

### CoV Isolates and Broad-range PCR Primer Pairs

Table 1 lists all the CoV used in this study. Multiple sequence alignments of all available CoV nucleotide sequences from GenBank were scanned to identify pairs of potential PCR priming loci. Two target regions were selected in CoV ORF-1b (annotations based on Snijder et al. [20]), 1 in RNA-dependent RNA polymerase (RdRp) and the other in Nsp14 (Table 2). 5′ propynyl-modified pyrimidine nucleotides (shown in bold) were positioned at universally conserved positions within these primers to extend the breadth of broad-range priming to allow efficient PCR from all CoV species tested.

**Table 1:** Coronaviruses used in the study and mass spectrometry results*

Group	CoV species	Strain	Source	Strand	RdRp		Nsp14	
					Experiment determined masses (Da)	Calculated base compositions	Experiment determined masses (Da)†	Calculated base compositions
1	Canine	1-71	VR809	S	27486.514	A24 G24 C8 T32	42475.955	A33 G31 C19 T54
				AS	26936.574	A32 G8 C24 T24	42185.117	A54 G19 C31 T33
		CCV-TN449	VR2068	S	27471.510	A24 G24 C9 T31	42474.899	A34 G30 C18 T55
				AS	26952.548	A31 G9 C24 T24	42184.072	A55 G18 C30 T34
	Feline	WSU 79-1683	VR-989	S	27471.517	A24 G24 C9 T31	42490.945	A33 G31 C18 T55
				AS	26952.556	A31 G9 C24 T24	42169.118	A55 G18 C31 T33
		DF2	VR2004	S	27472.497	A23 G25 C10 T30	42450.904	A33 G30 C19 T55
				AS	26953.536	A30 G10 C25 T23	42209.081	A55 G19 C30 T33
	Human 229E	229E	VR740	S	27450.532	A25 G24 C11 T28	42462.994	A36 G30 C20 T51
				AS	26975.545	A28 G11 C24 T25	42198.061	A51 G20 C30 T36
		229E	NHRC‡	S	27450.506	A25 G24 C11 T28	42462.930	A36 G30 C20 T51
				AS	26975.512	A28 G11 C24 T25	42198.040	A51 G20 C30 T36
2	Bovine	Calf diarrheal virus	VR874	S	27358.452	A22 G22 C12 T32	42606.039	A38 G32 C15 T52
				AS	27066.586	A32 G12 C22 T22	42052.897	A52 G15 C32 T38
	Human OC43	OC43	NHRC‡	S	27328.473	A22 G22 C14 T30	42580.959	A38 G31 C15 T53
				AS	27098.562	A30 G14 C22 T22	42076.028	A53 G15 C31 T38
	Murine hepatitis virus	MHV1	VR261	S	27344.491	A21 G23 C14 T30	42602.022	A37 G34 C18 T48
				AS	27083.564	A30 G14 C23 T21	42061.016	A48 G18 C34 T37
		JHM-thermostable	VR1426	S	27344.497	A21 G23 C14 T30	42529.960	A34 G34 C21 T48
				AS	27083.571	A30 G14 C23 T21	42136.047	A48 G21 C34 T34
		MHV-A59	VR764	S	27344.503	A21 G23 C14 T30	42599.989	A34 G35 C18 T50
				AS	27083.572	A30 G14 C23 T21	42064.089	A50 G18 C35 T34
	Rat	8190	VR1410	S	27344.491	A21 G23 C14 T30	42544.967	A34 G34 C20 T49
				AS	27083.567	A30 G14 C23 T21	42120.041	A49 G20 C34 T34
3	Infectious bronchitis virus	Egg-adapted	VR22	S	27396.544	A24 G24 C14 T26	42530.984	A33 G32 C17 T55
				AS	27032.524	A26 G14 C24 T24	42129.100	A55 G17 C32 T33
4	SARS	TOR2	University of Manitoba§	S	27298.518	A27 G19 C14 T28	42519.906	A34 G33 C20 T50
				AS	27125.542	A28 G14 C19 T27	42144.026	A50 G20 C33 T34
		Urbani	CDC¶	S	27298.518	A27 G19 C14 T28	42519.906	A34 G33 C20 T50
				AS	27125.542	A28 G14 C19 T27	42144.026	A50 G20 C33 T34

*CoV, coronavirus; SARS, severe acute respiratory syndrome. †Exact mass measurements for the sense and antisense strands of the dsDNA amplicon reported. Experimentally observed masses were within ±1ppm of expected masses, based on sequence data for each of the amplified DNA. Sense and antisense strand base compositions reported. ‡Clinical isolate obtained from Kathryn Holmes, University of Colorado, via Kevin Russell, Naval Health Research Center, San Diego. §Obtained from Heinz Feldmann, University of Manitoba. ¶Obtained from Dean Erdman, Centers for Disease Control and Prevention.

**Table 2:** PCR primer pairs used in this study*

Primer name	Gene name	Product name	Genome coordinates	Orientation	Product length (bp)	Sequence (5′ to >3′)
RdRp primer	ORF 1b	Nsp12-pp1ab (RdRp)	15146–15164	Sense	88	TAAG**TT**T**T**ATGGCGGCTGG
			15213–15233	Antisense		TTTAGGATAGT**CCC**AACCCAT
Nsp14 primer	ORF 1b	Nsp14-pp1ab (nuclease ExoN homolog)	19113–19138	Sense	137	TGTTTG**TTTT**GGAATTGTAATGTTGA
			19225–19249	Antisense		TGGAATGCATGC**TT**A**TT**AACATACA

*All coordinates are based on SARS TOR2 genome (GenBank accession no. NC_004718.3). 5′ propynyl-modified pyrimidine nucleotides are shown in **bold**. Each primer was designed to include a thymidine (T) nucleotide on the 5′ end to minimize addition of nontemplated adenosine (A) during polymerase chain reaction (PCR) (data not shown). RdRp, RNA-dependent RNA polymerase.

For each primer region, a database of expected base compositions (A, G, C, and T base counts) from all known CoV sequences in GenBank was generated (data not shown) and used in the identification and classification of the test isolates. Several of the isolates used in this study did not have a genome sequence record in GenBank. Experimentally measured base compositions from these isolates were independently verified by sequencing ≈500 base pair (bp) regions that flanked both target regions used in this study (GenBank accession nos.AY874541 and AY878317-AY878324).

### RNA Extraction, Reverse Transcription, and PCR

RNA was isolated from 250 μL of CoV-infected cells or culture supernatant spiked with 3 μg of sheared poly-A DNA using Trizol or Trizol LS, respectively (Invitrogen Inc., Carlsbad, CA, USA) according to the manufacturer’s protocol. Reverse transcription was performed by mixing 10 μL of the purified RNA with 5 μL of water treated with diethyl pyrocarbonate (DEPC, Sigma-Aldrich Co., St. Louis, MO, USA) containing 500 ng random primers, 1 μg of sheared poly-A DNA, and 10 U SUPERaseIn (Ambion Inc., Woodlands, TX, USA). The mixture was heated to 60°C for 5 min and then cooled to 4°C. Following the annealing of the random primers to the RNA, 20 μL of first-strand reaction mix consisting of 2x first-strand buffer (Invitrogen Inc.), 10 mmol/L DTT, 500 µmol/L deoxynucleoside triphosphates (dNTPs), and 7.5 U SuperScript II was added to the RNA primer mixture. The RNA was reversed transcribed for 45 min at 45°C. Various dilutions of the reverse transcription reaction mixes were used directly in the PCR reactions.

All PCR reactions were performed in 50 μL with 96-well microtiter plates and M.J. Dyad thermocyclers (MJ Research, Waltham, MA, USA). The PCR reaction buffer consisted of 4 U Amplitaq Gold (Applied Biosystems, Foster City, CA USA), 1x buffer II (Applied Biosystems, Foster City, CA, USA), 2.0 mmol/L MgCl_2_, 0.4 mol/L betaine, 800 μmol/L dNTP mix, and 250 nmol/L propyne-containing PCR primers. The following PCR conditions were used to amplify CoV sequences: 95°C for 10 min followed by 50 cycles of 95°C for 30 s, 50°C for 30 s, and 72°C for 30 s. After PCR, the amplified products were desalted before analysis by electrospray ionization Fourier transform ion cyclotron resonance mass spectrometry (ESI-FTICR-MS) by methods described previously (*21*). A small oligonucleotide SH2 (CGTGCATGGCGG, Synthetic Genetics, San Diego, CA, USA) was added as an internal mass standard (*22*); the final concentration of SH2 was 50 nmol/L.

### Mass Spectrometry and Signal Processing

The mass spectrometer used in this work is based on a Bruker Daltonics (Billerica, MA, USA) Apex II 70e ESI-FTICR-MS that used an actively shielded 7 Tesla superconducting magnet. All aspects of pulse sequence control and data acquisition were performed on a 1.1 GHz Pentium II data station running Bruker’s Xmass software (Bruker Daltonics). Inputs to the signal processor are the raw mass spectra for each of the parallel PCR reactions used to analyze a single sample. The ICR-2LS software package (*23*) was used to deconvolute the mass spectra and calculate the mass of the monoisotopic species using an “averagine” fitting routine (*24*) modified for DNA (Drader et al., unpub. data). Using this approach, monoisotopic molecular weights were calculated. The spectral signals were algorithmically processed to yield base composition data as described previously (*25*). The amplitudes of the spectra are calibrated to indicate the number of molecules detected in the mass spectrometer; m/z and the m/z values are corrected by using internal mass standards. The algorithm computes the organism’s identity and abundances consistent with observations over all the PCR reactions run on the input sample.

## Results and Discussion

### Detection of Individual CoV Isolates

**Figure 1:**
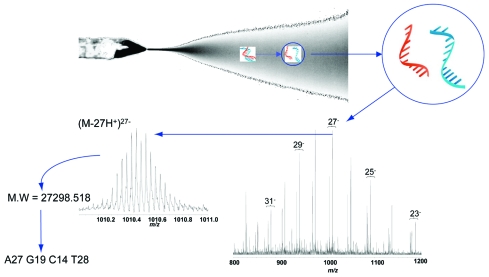
Electrospray ionization Fourier transfer ion cyclotron resonance (ESI-FTICR) mass spectrum from the polymerase chain reaction (PCR) amplicons from the severe acute respiratory syndrome (SARS)-associated coronavirus obtained with the propynylated RNA-dependent RNA polymerase primer pairs. The electrospray ionization conditions separate the sense and antisense strands of the PCR products. Multiple charge states are observed across the m/z range shown. The inset shows an expanded view of the isotope envelope of the (M-27H+)27- species. As enumerated in Table 1, the derived molecular masses for the amplicon strands are 27298.518 (+ 0.03) Da and 27125.542 (+ 0.03) Da, corresponding to an unambiguous base composition of A27G19C14T28/ A28G14C19T27 for the double-stranded amplicon, the composition expected for the SARS isolate.

For broad-range detection of all CoV, the 2 PCR primer target regions shown in Table 2 were used against each virus listed in Table 1. Resultant products were desalted and analyzed by FTICR-MS by methods described previously (*21*). The spectral signals were algorithmically processed to yield base composition data. Figure 1 shows a schematic representation of electrospray ionization, strand separation, and the actual charge state distributions of the separated sense and antisense strands of the PCR products from the RdRp primer pair for SARS-CoV. Due to the accuracy of FTICR-MS (mass measurement error ± 1 ppm), all detected masses could be unambiguously converted to the base compositions of sense and antisense strands (*25*).

One of the limitations of all molecular methods for detecting pathogens, including the one described here, is that unexpected variations in PCR primer target sequences in unknown species can lead to missed detection. To minimize this possibility, the primers designed in this study were selected on the basis of highly conserved regions identified by multiple sequence alignments of all previously known CoV species sequences. Further, we chose 2 amplification targets for redundant detection of the CoV and to have increased resolution to distinguish the different viral species. Both primer pairs were tested against multiple isolates from the 3 previously known CoV species groups and from SARS-CoV isolates.

The results from analysis of 14 CoV isolates are shown in Table 1. For both target regions, the measured signals agreed with compositions expected from the known CoV sequences in GenBank (data not shown). Several of the isolates used in this study did not have a genome sequence record in GenBank. Nevertheless, we were able to amplify all test viruses and experimentally determine their base compositions. These experimentally determined base compositions were confirmed by sequencing (data not shown). Thus the strategy described here enables identification of organisms without the need for prior knowledge of the sequence, provided that the broad range primers do not fail to amplify the target because of excessive numbers of mismatches.

### Multiple CoV Isolates in Mixture

**Figure 2:**
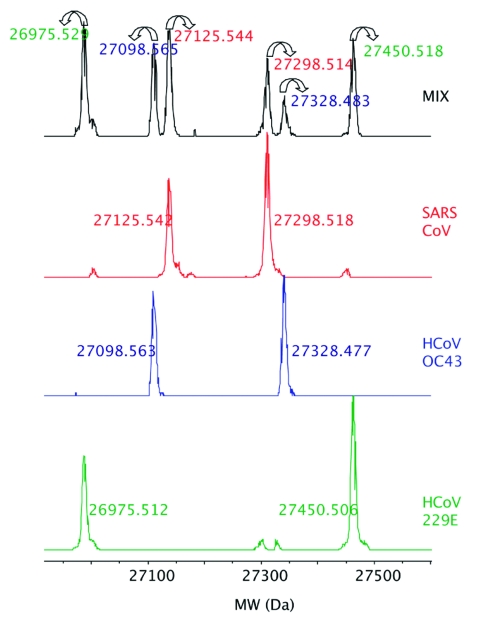
Detection of 3 human coronavirus (CoV) in a mixture. The deconvoluted (neutral mass) mass spectra obtained for the RNA-dependent RNA polymerase primer for the 3 human CoV, HCoV-229E, HCoV-OC43, and severe acute respiratory syndrome–associated CoV, which were tested individually and in a mixture are shown. Forward and reverse amplicons are shown with the measured monoisotopic masses for each strand. Colors of the monoisotopic masses for the mixed spectra correspond to the individual viral species.

To demonstrate the potential to detect multiple viruses in the same sample, as might occur during a coinfection, we pooled the viral extracts from 3 human CoV, HCoV-229E, HCoV-OC43, and SARS-CoV, and analyzed the mixture. Signals from all 3 viruses were clearly detected and resolved in the mass spectra (Figure 2), which demonstrated that coinfections of >1 CoV species could be identified. We have previously determined that the system can reliably detect multiple species in ratios of ≈1:1000, while varying input loads from 10 to 10,000 organisms (data not shown).

### Sensitivity

To determine sensitivity in a clinical sample, viable, titered SARS-CoV was added to human serum and analyzed in 2 ways. In the first, RNA was isolated from serum containing 2 concentrations of the virus (1.7 x 10^5^ and 170 PFU/mL), reverse transcribed to cDNA with random primers, and serially diluted (10-fold), before PCR amplification with both RdRp and Nsp14 primer sets. By using this approach, the assay was sensitive to ≈10^2^ PFU per PCR reaction (≈1.7 PFU/mL serum). We estimated the number of reverse-transcribed SARS genomes by competitive, quantitative PCR with a nucleic acid internal standard (data not shown). Analysis of ratios of mass spectral peak heights of titrations of the internal standard and the SARS cDNA showed that ≈300 reverse-transcribed viral genomes were present per PFU, similar to the ratio of viral genome copies per PFU previously reported for RNA viruses (*26*). By using this estimate, PCR primers were sensitive to 3 genome equivalents per PCR reaction, which is consistent with previously reported detection limits for optimized SARS-specific primers (*27*,*28*). In the second method, we spiked 10-fold dilutions of the SARS virus into serum before RT-PCR and could reliably detect 1 PFU (≈300 genomes) per PCR reaction or 170 PFU (5.1 x 10^4^ genomes) per mL serum. The discrepancy between the detection sensitivities in the 2 experimental protocols described above suggests that losses were associated with RNA extraction and reverse transcription when very little virus was present (<300 genome copies) in the starting sample in serum. This finding is consistent with results for direct measurement of RNA viruses from patient samples (*26*). Therefore, in a practical experimental analysis of a tissue sample, the limit of sensitivity was ≈1 PFU per PCR reaction.

### RNA Virus Classification with Base Compositions

We have described a novel approach using base composition analysis for viral identification. However, since RNA virus nucleotide sequences mutate over time within the functional constraints allowed by selection pressure (*29*), the utility of this method to correctly classify RNA viruses depends on the resolution needed for a particular application. We considered 2 specific applications. The first was to distinguish SARS-CoV from other species of CoV that infect humans, namely HCoV-OC43 and HCoV-229E. The second application was the utility of the technique for exploration of animal reservoirs for the discovery of SARS-related CoV species.

**Figure 3:**
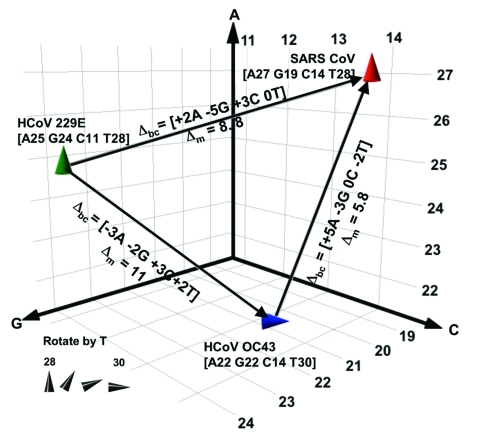
Spatial representation of base compositions for the 3 coronavirus (CoV) species known to infect humans. Severe acute respiratory syndrome (SARS), HCoV-OC43, and HCoV-229E base compositions in the region amplified by RNA-dependent RNA polymerase primers (Table 1) are plotted on the A, G, and C axes. T counts are shown by the tilt of the symbol. Within a species, all known isolates of each virus (37 isolates for SARS, 4 for HCoV-229E, and 2 for OC43) had identical sequences in this region. Δ_bc_ represents the number of changes in the A, G, C, and T bases needed for 1 species to be misidentified as another in the direction of the arrow. Δ_m_ represents the pairwise mutation distance between 2 species, or the cumulative probability of Δ_bc_ occurring.

To quantitatively analyze the resolving power of base compositions, we mathematically modeled base composition variations using known sequences of multiple isolates of hepatitis C virus (HCV) in GenBank (H. Levene, et al., unpub. data). HCV sequence-derived mutation probabilities were used to estimate the extent of base composition variations for CoV species. Figure 3 shows a plot of the base compositions for the RdRp target region for the 3 CoV known to infect humans. Δ_bc_ represents the net changes in composition required for strain variants of 229E or OC43 to be misidentified as SARS, and Δ_m_ the probability of occurrence of these changes. The cumulative probability of misclassifying either 229E or OC43 as SARS by using base composition measurements from both target regions was low (Δ_m_ >10), even allowing for unseen variations in those 2 viruses. Thus, for use in human clinical diagnostics, base composition analysis of the 2 target regions described here would provide corroborative information and accurate species identification of CoV infections.

**Figure 4:**
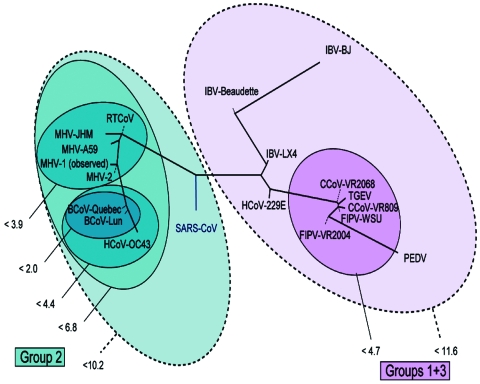
Representation of the mutational distances calculated for the 2 selected primer sets overlaid on the coronavirus phylogenetic tree. Each oval represents grouping of members contained within it; numbers next to the group indicate the maximum distance between any 2 members of the group. Distances are computed as the base 10 logarithm of the geometric average of the pair-wise probabilities for any given pair of base compositions.

To determine the utility of base composition analysis in the search for animal CoV species, we calculated the cumulative mutation distances for both target regions for all known CoV and plotted groups where all members fall within certain probability thresholds, as shown in Figure 4. A series of nested ovals represents subgroupings of species, where the maximal distance between known members of a subgroup is represented by the Δ_m_ next to the oval. By using the above classification metric, SARS-CoV would be considered the first member of a new group of CoV, not a member of the core group 2 cluster, although it would be placed closest to group 2 (Δ_m_ < 10.2). These findings are similar to those recently described by Snijder et al., who used sequence data from the replicase genes (5,487 bp) in ORF1b and suggested that the SARS-CoV was most closely related to and possibly an early split-off from group 2 CoV (*20*). However, substantial space exists around SARS-CoV where as yet undiscovered SARS-CoV could populate a subgroup without being confused with the group 2 or other CoV.

## Conclusion

The strategy we describe allows rapid identification of new viral species members of previously characterized viral families, without the need for prior knowledge of their sequence, through use of integrated electrospray ionization mass spectrometry and base composition analysis of broad-range PCR products. Broad-range PCR reactions are capable of producing products from groups of organisms, rather than single species, and the information content of each PCR reaction is potentially very high. Further, in many cases, including the SARS-CoV detection described in this article, priming across broadly conserved regions provides adequate species detection and taxonomic resolution. In cases where additional subspecies level classification becomes important, broad primers can be followed up with more species-specific primers that can detect even single nucleotide changes (SNPs) or alternatively, larger regions of the identified species can be analyzed by sequencing. Despite the advances in high throughput sequencing, however, it is impractical as a front-end detector in a routine survey and detection setting. The mass spectrometer is capable of analyzing complex PCR products at a rate of ≈1 minute per sample. Because the process is performed in an automated, microtiter plate format, large numbers of samples can be examined (>900 PCR reactions/day/instrument), which makes this process practical for large-scale analysis of clinical or environmental surveillance samples in public health laboratory settings. The current generations of ESI mass spectrometers used in the detector cost approximately U.S.$150,000 and can be operated 24 hours per day by trained technicians. Tools for analyzing mass spectrometry data are widely available and are described in detail elsewhere (*23*–*25*). A comparable alternative to the methods described here are microarrays, which can also provide broad range detection.

This approach can be extended to other viral, bacterial, fungal, or protozoal pathogen groups and is a powerful new paradigm for timely identification of previously unknown organisms that cause disease in humans or animals and for monitoring the progress of epidemics.
